# Bin1 targeted immunotherapy alters the status of the enteric neurons and the microbiome during ulcerative colitis treatment

**DOI:** 10.1371/journal.pone.0276910

**Published:** 2022-11-02

**Authors:** Sunil Thomas, Giancarlo Mercogliano, George C. Prendergast

**Affiliations:** 1 Lankenau Institute for Medical Research, Wynnewood, PA, United States of America; 2 Lankenau Medical Center, Wynnewood, PA, United States of America; University of Arkansas Fayetteville, UNITED STATES

## Abstract

Ulcerative colitis (UC) is a common chronic disease of the large intestine. Current anti-inflammatory drugs prescribed to treat this disease have limited utility due to significant side-effects. Thus, immunotherapies for UC treatment are still sought. In the DSS mouse model of UC, we recently demonstrated that systemic administration of the Bin1 monoclonal antibody 99D (Bin1 mAb) developed in our laboratory was sufficient to reinforce intestinal barrier function and preserve an intact colonic mucosa, compared to control subjects which displayed severe mucosal lesions, high-level neutrophil and lymphocyte infiltration of mucosal and submucosal areas, and loss of crypts. A dysbiotic microbiome may lead to UC. We determined the effects of Bin1 mAb on the gut microbiome and colonic neurons and correlated the benefits of immunotherapeutic treatment. In the DSS model, we found that induction of UC was associated with disintegration of enteric neurons and elevated levels of glial cells, which translocated to the muscularis at distinct sites. Further, we characterized an altered gut microbiome in DSS treated mice associated with pathogenic proinflammatory characters. Both of these features of UC induction were normalized by Bin1 mAb treatment. With regard to microbiome changes, we observed in particular, increase in *Enterobacteriaceae*; whereas *Firmicutes* were eliminated by UC induction and Bin1 mAb treatment restored this phylum including the genus *Lactobacillus*. Overall, our findings suggest that the intestinal barrier function restored by Bin1 immunotherapy in the DSS model of UC is associated with an improvement in the gut microbiome and preservation of enteric neurons, contributing overall to a healthy intestinal tract.

## Introduction

The major inflammatory bowel disease of the colon is Ulcerative Colitis (UC). Mucosal inflammation of the colon is the hallmark of UC. UC usually presents with bloody diarrhea that is diagnosed by histology and colonoscopy [1]. Patients with UC has a risk of developing colorectal cancer. The colorectal cancer risk is as high as 18% with 30 years of UC [2]. The increased risk of colorectal cancer is due in part to the loss of barrier function due to inflammation. As yet there are no fully effective drugs and therapies to protect against UC. Elevated levels of the inflammatory cytokines, tumor necrosis factor (TNF)‐α, and IFN‐γ is a hallmark of UC. While immune suppressors and anti-inflammatory drugs (eg, TNF‐α inhibitors) are currently prescribed for UC treatment, side effects including risk of opportunistic infections and the lack of efficacy in certain patients limit the quality of treatment [3]. Hence, there is a need in developing additional immunotherapies targeting the intestinal barrier so as to provide protection against UC.

The bridging integrator 1 (Bin1) (amphiphysin 2), is a nucleocytoplasmic adaptor protein with 10 isoforms. In 1996, the *BIN1* gene was initially identified as a tumor suppressor; subsequently, however in recent years, additional functions have been attributed to different protein transcripts. Bin1 is a member of the BAR (Bin‐Amphiphysin‐Rvs) family of adapter proteins. These proteins are implicated in diverse cellular processes including stress response, endocytosis, programmed cell death, actin organization, and transcriptional control. In late‐onset Alzheimerʼs disease, the *BIN*1 gene has recently been identified as the most important risk locus after apolipoprotein E [4].

Our group recently demonstrated that attenuation of *BIN1* gene reduced disease severity in a mouse model of experimental colitis occurring in association with an enhancement of epithelial barrier function. Based on the study, we explored whether the Bin1 monoclonal antibodies (mAb) developed by our laboratory phenocopied effects of genetic attenuation in the colitis model. We recently reported the development of a novel colitis therapy using both cell culture and animal models, targeting the Bin1 protein thereby supporting epithelial barrier function. DSS-induced colitis mice had severe lesions throughout the mucosa, high-level lymphocyte and neutrophil infiltration into the mucosal and submucosal areas, and loss of crypts; whereas animals treated with the cell penetrating Bin1 mAb protects against DSS-induced colitis by directly improving colonic epithelial barrier function that limit gene expression and cytokine programs that are associated with colonic inflammation [5, 6].

The interactions between the enteric nervous system (ENS) and the immune system play important roles in the modulation of inflammation. These interactions are mediated by neurotransmitters, neuromodulators, and cytokines that carry signals between enteric neurons and immune cells [7]. The ENS controls virtually all gastrointestinal (GI) functions including motility, secretion, blood flow, mucosal growth and aspects of the local immune system. It is clearly not understood whether the persistent alterations in gut function observed following inflammation are due to altered properties of enteric nerves [8].

A dysbiotic microbiome, inflammation and altered enteric neurons in response to inflammation is a hallmark of ulcerative colitis. In this paper, we report progress in how Bin1 mAb treatment protects against DSS-induced colitis; specifically, it protects enteric neurons thereby preventing bowel movement dysfunction, and that it promotes formation of a healthy gut microbiome.

## Materials and methods

All experimental protocols were approved by the institutional animal care and use committee of Lankenau Institute for Medical Research and were in accordance with the guidelines of the National Institutes of Health. All methods were carried out in accordance with relevant guidelines and regulations.

### Bin1 monoclonal antibodies

We used the therapeutic 99D Bin1 monoclonal antibody for our study [4–6, 9]. The antibody 99D recognizes an epitope within the C-terminal Myc binding domain encoded by exon 13 [10]. 99D exhibited the ability to improve barrier function as demonstrated by *in vitro* experiments with human Caco-2 colon cells and animal models of UC [5, 6].

### Experimental colitis model system

For the study, we used a therapeutic animal model of UC. Animals were weighed before, during and after treatments. Briefly, male mice (C57BL/6) of 5 weeks of age were fed with 3% dextran sodium sulfate (DSS, Alfa Aesar, MW 40kDa) in drinking water ad libitum (n = 5 per group). After 7 days of DSS treatment, mice were provided with distilled water. 24 hours after feeding with water they were injected i.p. (0.5 mg of purified antibody per mouse) with Bin1 mAb or antibody isotype control [5, 6]. After 7 days of Bin1 mAb or isotype control treatments, mice were euthanized with CO_2_ inhalation, and colons were measured and inspected for necropsy for gross macroscopic lesions. All methods in animals were performed in accordance with ARRIVE guidelines.

### Immunohistochemistry

The colon tissues taken from mice after DSS treatment or after treatment with Bin1 mAb were washed in PBS and were processed for immunohistochemistry according to Thomas et al. (2014) [11]. The tissues were probed with antibodies for neuronal nuclear protein (NeuN), glial fibrillary acidic protein (GFAP) (Cell Signaling, MA), protein gene product 9.5 (PGP9.5) (Invitrogen/ThermoFisher), microtubule-associated protein 2 (MAP2) (Cell Signaling, MA), neuron-specific class III beta-tubulin (TuJ1) (BioLegend, CA) according to the manufacturer’s instructions. The cells were mounted using Fluoromount-G (Southern Biotech, AL) and visualized by confocal microscopy (Nikon Eclipse TI, Japan). Images were taken from different fields from the same slide. The experiments were repeated thrice. The corrected total cell fluorescence was analyzed using Image J, the open-source image analysis software.

### Analyses of the gut microbiome

The mice were placed on a raised platform to determine the quantity of fecal pellets and urine excreted in 10 minutes. We could collect 4 to 8 fecal pellets in 10 minutes from untreated control mice. Hence, 10 minutes was maintained to monitor the fecal pellets in the treatment groups. Placing the animals on a raised platform improved fecal pellet collection compared to leaving the animals in a container for the same duration. The microbiome of the fecal pellet from the animals were analyzed directly or they were cultured in a biosimulator in Luria-Bertani (LB) medium in aerobic and anaerobic conditions. The biosimulator induce proliferation of microorganisms [12]. The microorganisms were cultured for 48 hours in the biosimulator, and the contents pelleted in a centrifuge at 5000 g. Microbial DNA extraction was extracted from samples by using DNeasy PowerSoil Kit–(QIAGEN) following directions of the manufacturer. The samples were subjected to 16S rRNA sequencing (Arizona State University Microbiome Core) for taxonomic identification. The experiments were repeated three times.

### Microbiome library preparation methodology

Bacterial community analysis was performed via next generation sequencing in MiSeq Illumina platform. Amplicon sequencing of the V4 region of the 16S rRNA gene was performed with the barcoded primer set 515f/806r designed by Caporaso et al. (2011) [13] and following the protocol by the Earth Microbiome Project (EMP) (http://www.earthmicrobiome.org/emp-standard-protocols/) for the library preparation. PCR amplifications for each sample are done in triplicate, then pooled and quantified using Quant-iT™ PicoGreen® dsDNA Assay Kit (Invitrogen). A no template control sample is included during the library preparation as a control for extraneous nucleic acid contamination. 240 ng of DNA per sample are pooled and then cleaned using QIA quick PCR purification kit (QIAGEN). The pool is quantified by Illumina library Quantification Kit ABI Prism^®^ (Kapa Biosystems). Then, the DNA pool is diluted to a final concentration of 4 nM then denatured and diluted to a final concentration of 4 pM with a 15% of PhiX. Finally, the DNA library was loaded in the MiSeq Illumina and run using the version 2 module, 2x250 paired-end, following the directions of the manufacturer.

### Microbiome downstream analysis

Downstream visualization and statistics were performed by the Harvard T.H. Chan School of Public Health Microbiome Analysis Core. Briefly, samples with read counts lower than 5,000 post filtering, denoising, merging, and chimera removal were excluded from downstream analysis. Phenotypic variables were tested against the bacterial communities’ alpha and beta diversity metrics, InvSimpson and Bray-Curtis dissimilarity and unweighted and weighted UniFrac distance, respectively. Alpha diversity was calculated using the estimate richness function in phyloseq [14] and differences in diversity were found using an ANOVA test on linear models, both univariable and multivariable, and box plots were used to visualize trends. Beta diversity was calculated using the vegan [15] package in R and significant differences in community composition were tested using an omnibus univariable PERMANOVA test using the adonis function within the vegan package in R. Next, we incorporated covariates in multivariate models. Further, principal coordinates analysis (PCoA) plots were created on the Bray-Curtis dissimilarities and unweighted and weighted UniFrac distances. To display the samples’ community compositions, stacked bar plots and heat maps on taxonomic relative abundances were constructed on the top 30 taxa and annotated with the phenotypes. All diversity trends and community composition visualizations were created using the ggplot2 package in R [16].

Per-feature differences in the microbial composition were explored with the MaAsLin2 [17] tool, which tests for statistically significant associations determined by testing each clade in a hierarchical manner after normalization from counts to relative abundances and log transforming these data. Among each of the comparisons generated, multiple comparisons are adjusted using a Benjamini and Hochberg correction and FDR corrected p-values of 0.25 or lower are reported as significant. Thus, MaAsLin2 identifies microbial organisms that reach a statistically significant association with each of the phenotypes. For all analyses other than alpha diversity, feature tables were filtered requiring a microbial feature to have at least 0.01% relative abundance in at least 10% of all samples.

### Statistics

Unpaired two‐tailed Student t tests were used to compare sets of data obtained from independent groups. Statistical significance was considered at the P<0.05 level.

## Results

### Phenotypic changes in mice after treatment

**Fig 1:**
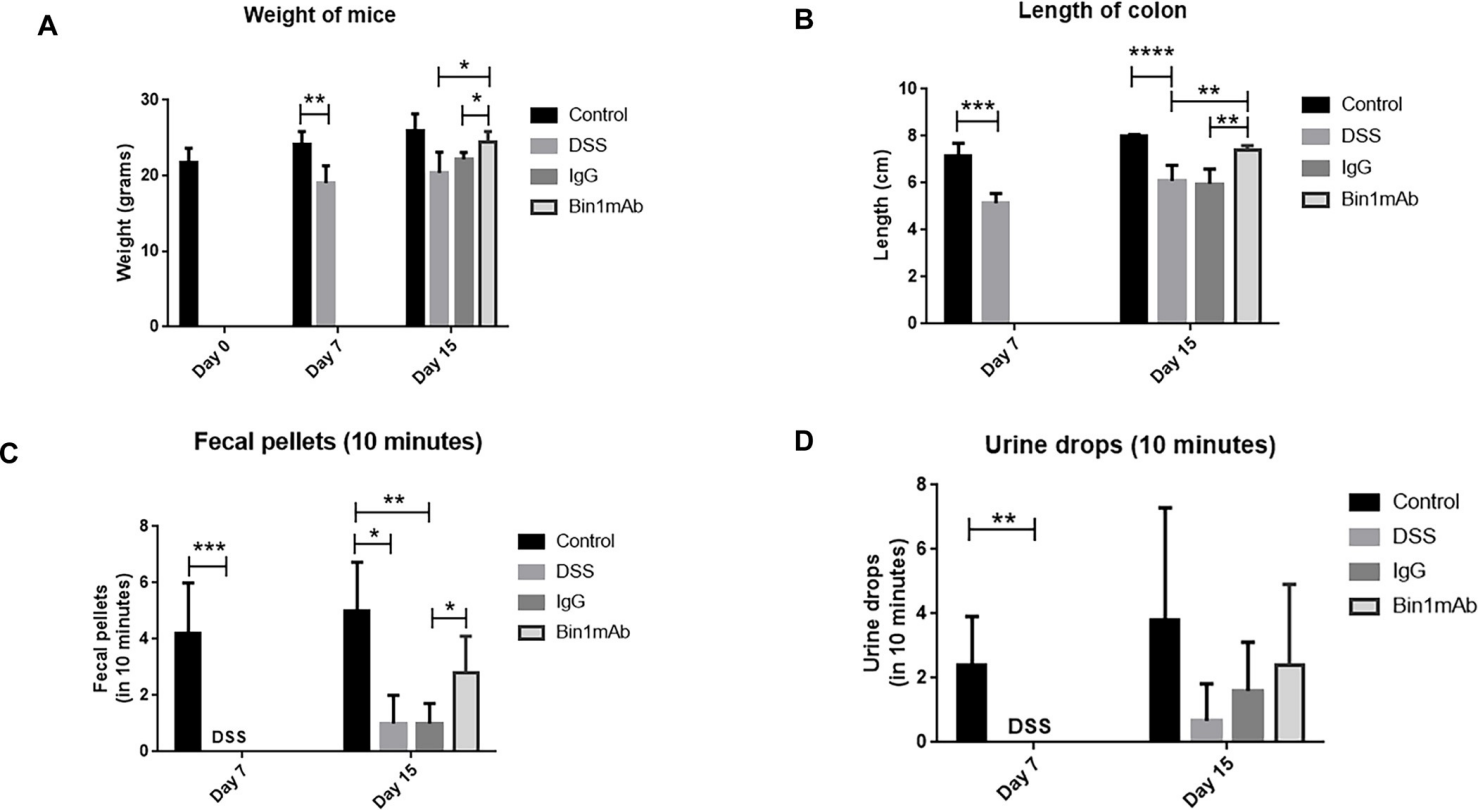
Phenotypic effects of Bin1 mAb treatment in DSS-treated mice. There was a change in (A) weight of mice, (B) length of colon, (C) the amount of fecal pellets, and (D) urine drops after treatment with Bin1 mAb after subjecting to DSS treatment (*P<0.05, **P<0.01, ***P<0.001, ****P<0.0001, as determined by t test). All results are expressed as Mean ± SD; (n = 5 mice per treatment). **Fig 1A.** Day 7: Control and DSS *p<0.0038. Day 15: DSS and Bin1 mAb *p<0.0285, IgG and Bin1mAb *p<0.0156. **Fig 1B.** Day 7: Control and DSS *p<0.0002, Day 15: Control and DSS *p<0.0005, DSS and Bin1 mAb *p<0.0057, IgG and Bin1 mAb *p< 0.0015. **Fig 1C.** Day 7: Control and DSS *p<0.0008. Day 15: Control and DSS *p<0.0116, Control and IgG *p<0.0265. **Fig 1D.** Day 7: Control and DSS *p<0.0076. Bin1, bridging integrator 1; DSS, dextran sodium sulfate; mAb, monoclonal antibodies.

The mice were provided with standard diet during the experiment. Equal amount of food and 3% DSS were provided to all the animals in the treatment groups. After 7 days of DSS consumption, mice were switched to regular water. Mice treated with DSS alone had decrease in weight compared to the controls (Fig 1A). Bin1 mAb treated mice had increased weight compared to the DSS treated counterpart. DSS influenced the length of the colon. The colon length was longer in the untreated mice and in the Bin1 mAb treated mice compared to the mice treated with DSS (Fig 1B).

DSS treated animals had no fecal pellets or urine after 10 minutes of monitoring on day 7. The fecal pellets and urine improved on day 15 in DSS treated animals. We demonstrated previously that Bin1 mAb immunotherapy protected against UC induction in the DSS model [5, 6]. In the present study, we determined that Bin1 mAb-treated animals had better counts of fecal pellets and urine compared to DSS-only or IgG-treated animals (Fig 1C and 1D). These data argued that Bin1 mAb treatment improved bowel health in animals where UC was induced.

### Changes in the enteric neurons and glial cells after induction of UC

**Fig 2:**
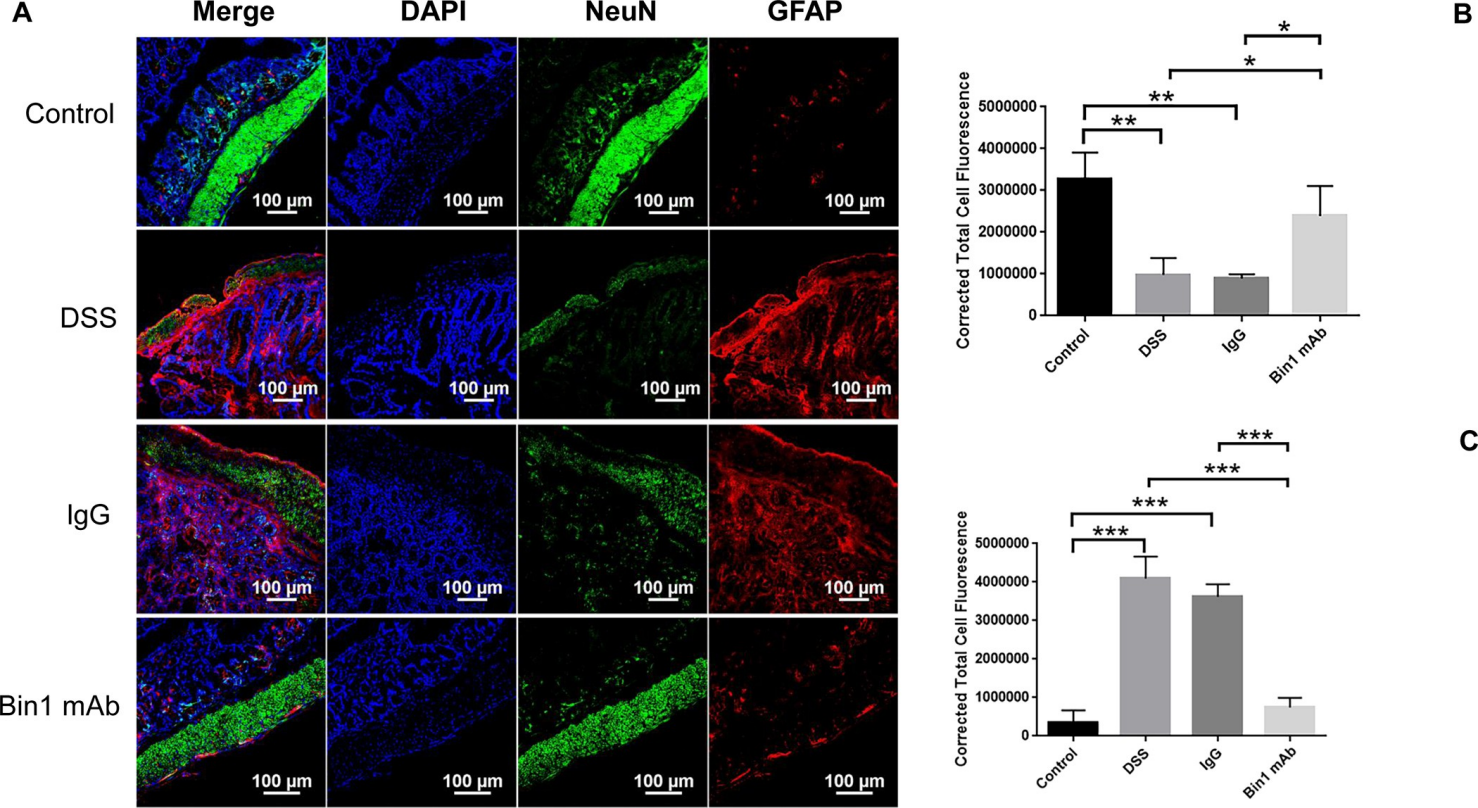
Bin1 mAb treatment improved NeuN expressing enteric neurons and GFAP expressing glial cells after DSS-induced colitis. **Fig 2A**. Effect of Bin1 mAb treatment on neuronal and glial abundance in the gut of DSS-induced colitis mice as determined by confocal microscopy. Bin1 mAb treated mice had high expression of NeuN and low expression of GFAP (n = 5). **Fig 2B**. Bin1 mAb treated mice had high levels of NeuN expressing neuronal cells in the muscularis of the colon as quantitated by CTCF analysis (*P<0.05, **P<0.01, as determined by t test). Control and DSS *p<0.0060, Control and IgG *p<0.0030, DSS and Bin1 mAb *p<0.0414, IgG and Bin1 mAb *p< 0.0234. **Fig 2C**. Bin1 mAb treated mice had low levels of GFAP expressing glial cells in the colon as quantitated by CTCF analysis (CTCF analysis, n = 3 mice tissues per treatment) (*P<0.05, **P<0.01, as determined by t test). Control and DSS *p<0.0006, Control and IgG *p<0.0002, DSS and Bin1 mAb *p<0.0007, IgG and Bin1 mAb *p<0.0003.

The enteric nervous system (ENS) is the intrinsic neural network of the gastrointestinal tract that orchestrates gastrointestinal behavior independently of the central nervous system (CNS). ENS dysfunction is often linked to digestive disorders. The activity of neurons during inflammation has profound consequences for gastrointestinal secretion and motility [7]. We determined the changes in the enteric neurons after DSS-induced colitis and during its treatment with Bin1 mAb. To determine the expression of enteric neurons and glial cells *in situ*, we analyzed these cells in the colon of mice by confocal microscopy. The colon of the untreated control and the Bin1 mAb treated mice had high expression of NeuN. Most of the enteric neurons were localized to the muscularis. The DSS-treated animals exhibited disintegration of enteric neurons, whereas IgG-treated animals exhibited low levels of enteric neurons (Fig 2). Conversely, the colon of DSS and IgG treated animals had higher expression of glial cells compared to the untreated controls or Bin1-mAb treated animals (Fig 2). The glial cells were mostly localized to the submucosa of the colon.

**Fig 3:**
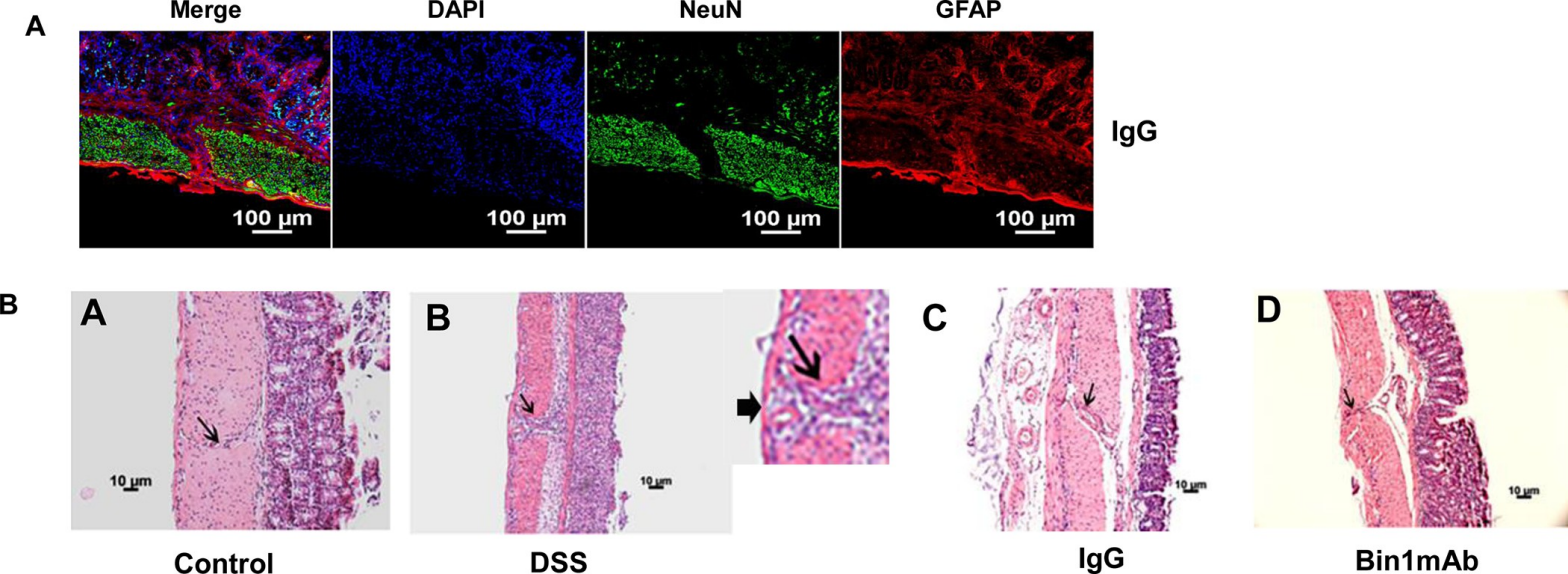
The muscularis of the colon is not a continuous entity. **Fig 3A.** The muscularis of the colon has openings (*anigma*) that transport glial cells (stained with GFAP), which protect the enteric neurons (n = 5). **Fig 3B.** H and E staining of the mouse colon showing that muscularis is not a continuous entity. The muscularis of the colon is punctuated with openings (*anigma*) (n = 5; representative image). Inset in figure B shows an intact serosa in the muscularis (large arrow) and *anigma* in the muscularis (long arrow).

The glial cells are localized in the submucosa and it is not known how they access the enteric neurons in the muscularis. The muscularis of the colon is not a continuous entity, rather it is innervated with arteries (arterial plexus) and nerves (myenteric plexus) [18]. It is not understood whether there are plexus free openings in the colon muscularis. Our confocal microscopy analysis revealed punctuated openings in the muscularis. Notably, glial cells were sighted in these regions such that they appeared to be crossing the openings of the muscularis into the serosa layer (Fig 3A). To confirm whether these openings are consistently present in the muscularis of the mouse, we observed the colon of mice in all the treatment groups by light microscopy after standard H&E histochemical staining. The openings were consistently seen in the muscularis of the colon in all treatment groups, arguing that it is not in response to a particular treatment (Fig 3B). The openings (*anigma;* Greek, opening) of the muscularis may enable the movement of glial cells to protect the enteric neurons.

**Fig 4:**
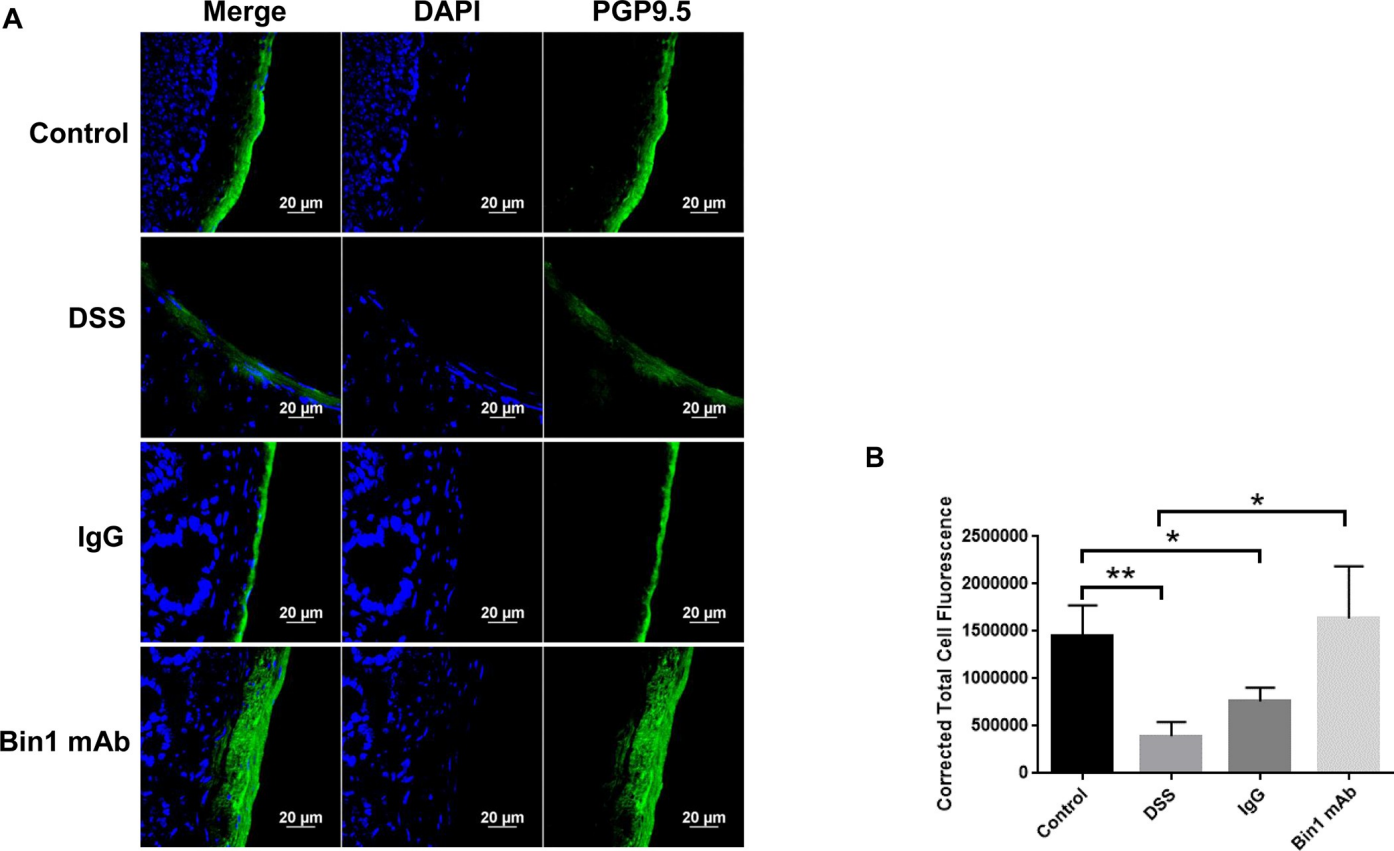
Treatment with Bin1 mAb improved PGP9.5 expressing enteric neurons after DSS-induced colitis. **Fig 4A**. Effect of Bin1 mAb treatment on the expression of PGP9.5 neuronal cells in the colon of DSS-induced colitis mice as determined by confocal microscopy. Representative image from two independent experiments shown. **Fig 4B**. Bin1 mAb treated mice had high levels of PGP9.5 expressing neuronal cells in the muscularis of the colon as quantitated by CTCF analysis (CTCF analysis, n = 3 mice tissues per treatment) (*P<0.05, **P<0.01, as determined by t test). Control and DSS *p<0.0063, Control and IgG *p<0.0259, DSS and Bin1 mAb *p<0.0197.

**Fig 5:**
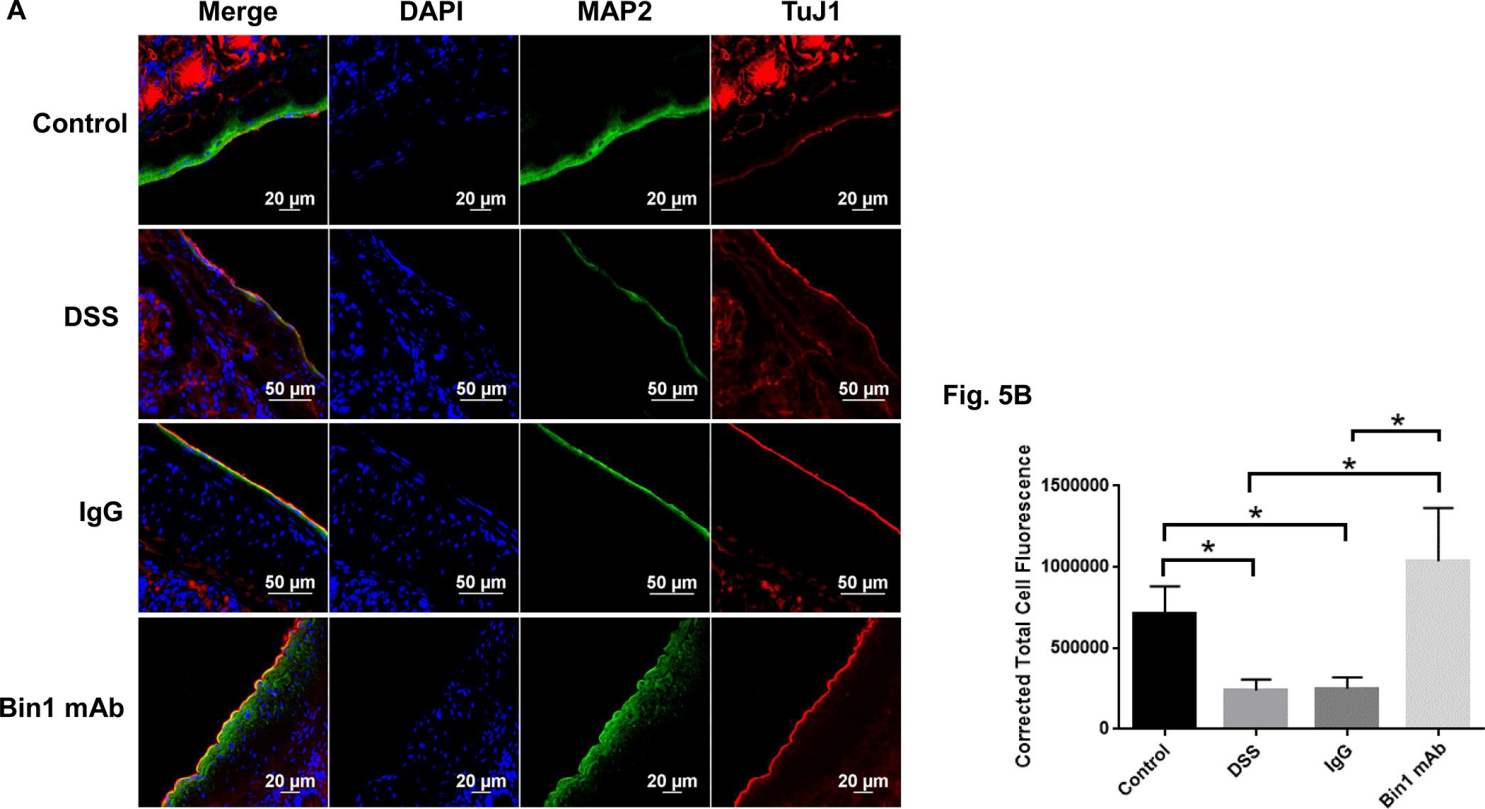
Bin1 mAb treatment improved MAP2 expressing enteric neurons after DSS-induced colitis. **Fig 5A**. Effect of Bin1 mAb treatment on the expression of MAP2 and TuJ1 neuronal cells in the colon of DSS-induced colitis mice as determined by confocal microscopy. Representative image from two independent experiments shown. **Fig 5B**. Bin1 mAb treated mice had high levels of MAP2 expressing neuronal cells in the muscularis of the colon as quantitated by CTCF analysis (CTCF analysis, n = 3 mice tissues per treatment) (*P<0.05 as determined by t test). Control and DSS *p<0.0112, Control and IgG *p<0.0124, DSS and Bin1 mAb *p<0.0144, IgG and Bin1 mAb *p<0.0152.

As the marker NeuN is not neuronal specific, we used the neuronal markers PGP9.5 and MAP2 for quantitation of the enteric neurons of the colon. DSS treatment, diminished the expression of PGP9.5 and MAP2 in the colon. However, treatment with Bin1 mAb significantly improved the expression of PGP9.5 and MAP2 in the colon demonstrating neurogenesis during immunotherapy (Figs 4 and 5). The IgG treatment did not improve neurogenesis of the ENS significantly. TuJ1 was localized to the colonic walls and the villi and there was no changes between treatments (Fig 5).

### Changes in the microbiome after induction of UC

It has been demonstrated that germ-free mice have an immature ENS that is normalized upon colonization with a normal microbiota. Communication between the microbiota and enteric neurons initiate serotonin release and activation of the serotonin (5-HT4) receptor. The interaction between the dysbiotic microbiota and ENS may lead to gastrointestinal disorders [19].

**Fig 6:**
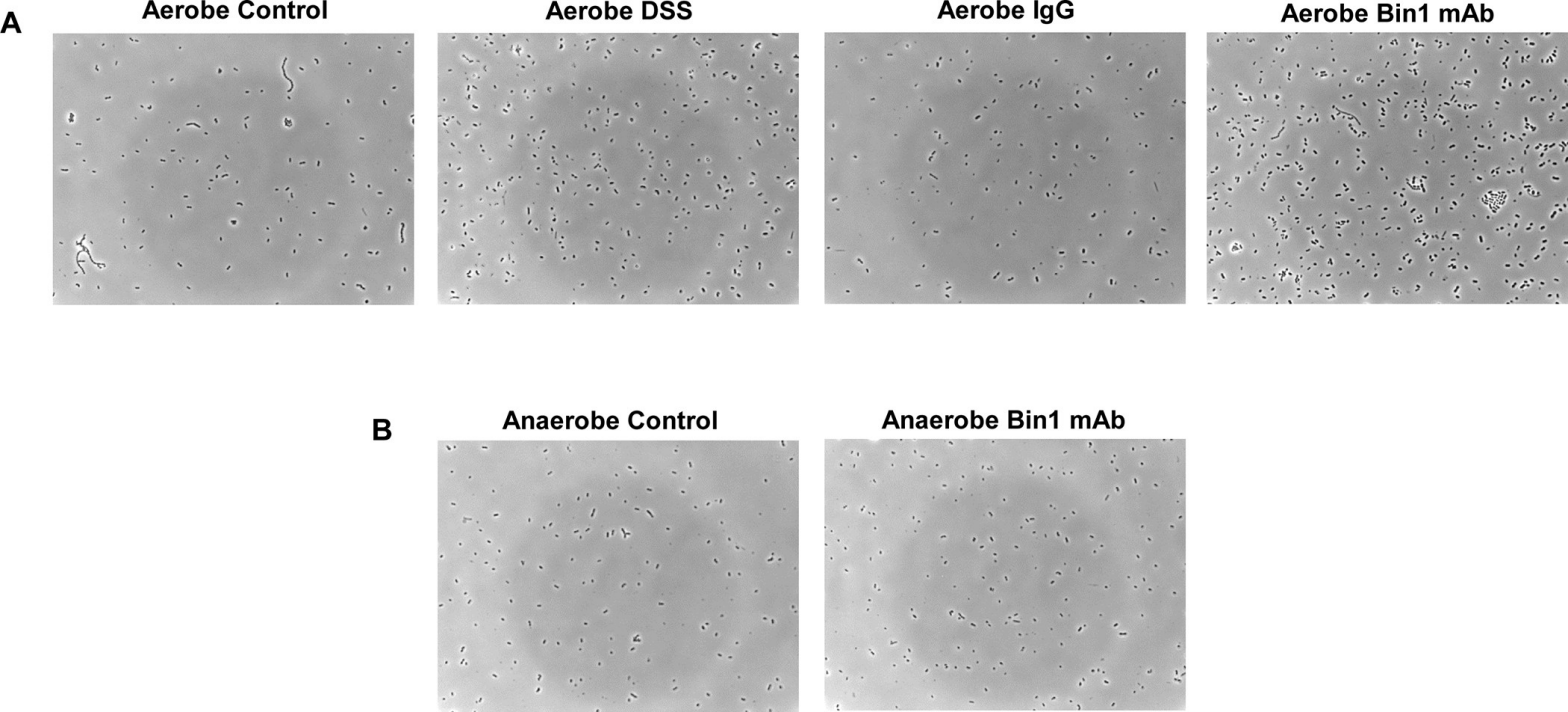
Morphological changes in bacteria of the gut after the treatment with Bin1 mAb. The fecal pellets were collected from untreated control mice, DSS-induced colitis mice, IgG treated mice and Bin 1 mAb treated mice; they were cultured aerobically and anaerobically and the morphology observed under phase contrast microscopy. **Fig 6A.** Aerobic culture of microorganisms show that the phenotype of Bin1 mAb treated mice has bacteria similar to the control (Phase contrast, 500X) (n = 5; representative image). **Fig 6B.** Anaerobic culture of microorganisms from fecal pellets were slow growing. There was no change in phenotype of the microorganisms between Bin 1 mAb treated and control groups (Phase contrast, 500X) (n = 5; representative image).

UC etiology is not understood. One of the hypotheses is that a dysbiotic microbiome is responsible for induction of UC [20]. In this study, the stool of mice before DSS-induced colitis, during DSS-induced colitis and during Bin1 mAb immunotherapy or control treatments was collected for microbiome analysis. We cultured the microbiome from fecal pellets obtained from experimental subjects in a biosimulator [12] under aerobic and anaerobic conditions. The bacteria had lower growth rate under anaerobic conditions. However, the bacteria multiplied rapidly under aerobic conditions. We observed the morphology of the bacteria under phase contrast microscopy. There was a drastic change in the morphology of the bacteria after the onset of DSS-induced colitis. The untreated control animals had a heterogenous population of bacteria. We observed single-celled and chain-like bacterial structures in the microbiome from untreated mice. However, the DSS treated animals had a homogenous single-celled bacterial morphology. There was no change in the morphology after treatment with IgG; whereas, treatment with the Bin1 mAb influenced the morphology of the microbiome. The morphology of the bacterial cells was similar to the untreated controls demonstrating that Bin1 mAb influences the microbiome during DSS-induced colitis treatment (Fig 6A and 6B).

**Fig 7:**
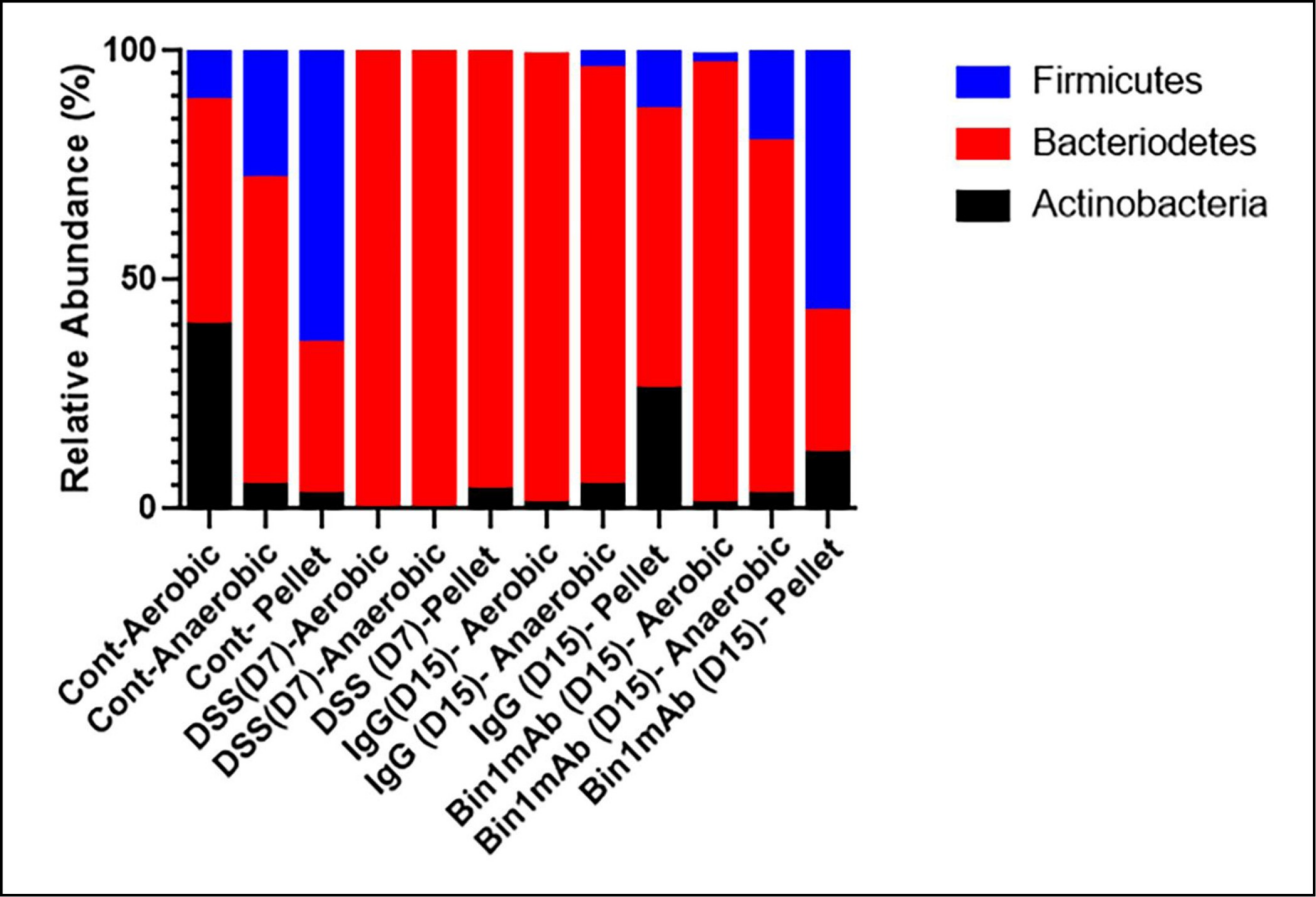
Microbiome sequencing by 16S rRNA showed lack of Firmicutes in the DSS-induced colitis mice in the fecal pellet or when cultured in a biosimulator under aerobic and anaerobic conditions. The fecal pellets were collected from untreated control mice, DSS-induced colitis mice (D7, Day 7), IgG treated mice (D15, Day 15) and Bin 1 mAb (D15, Day 15) treated mice; they were directly analyzed or cultured aerobically and anaerobically in a biosimulator. The level of Firmicutes improved during treatment with the Bin1 mAb (n = 3 per treatment).

**Fig 8:**
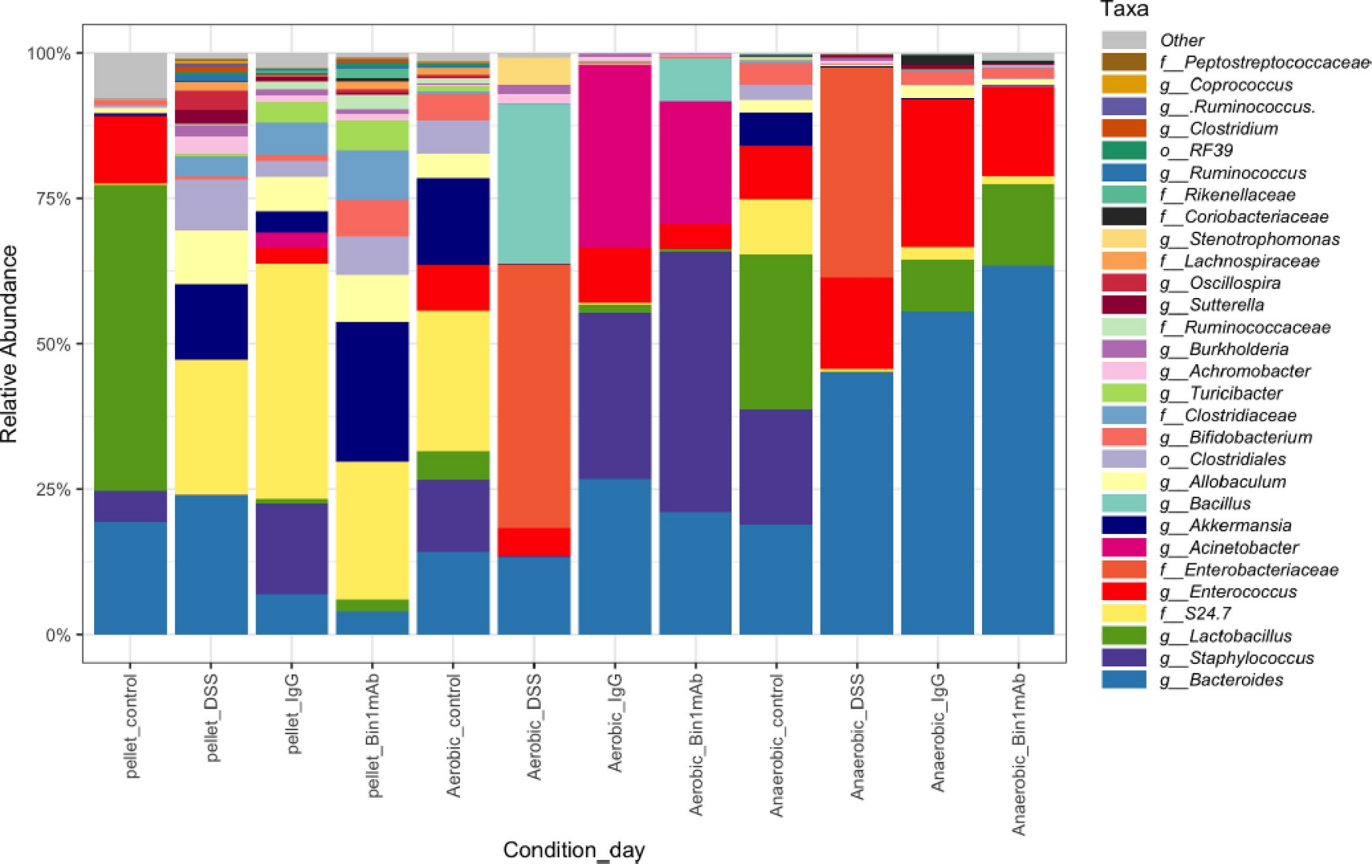
The relative abundance of the microbiome of the fecal pellets of mice from different treatment conditions. The bacteria of the genus *Lactobacillus* is totally absent in the UC mice. The UC mice is enriched in *Enterobacteriaceae*. (n = 3 mice per treatment).

Subsequently, we sequenced the microbiome by 16S rRNA to understand the taxonomy of the bacteria during DSS-induced colitis and its treatment. Notably, we observed a lack of Firmicutes during DSS-induced colitis in the fecal pellet or when cultured in a biosimulator under aerobic and anaerobic conditions. This reduction in Firmicutes was improved during treatment with the Bin1 mAb (Fig 7). *Enterobacteriaceae* were considerably increased during DSS-induced colitis. They were observed more when cultured aerobically or anaerobically rather than in the fecal pellet (Fig 8). Data from the microbiome sequencing showed that the bacteria of the genus Lactobacillus was totally absent in the DSS-induced colitis mice. *Lactobacillus* are predominantly anaerobes and levels of bacteria in this genus were elevated in subjects treated with the Bin1 mAb (Fig 8). These data indicated that the benefits of Bin1 mAb treatment in UC were associated with favorable changes in *Lactobacillus* consistent with potential involvement in maintaining gut health of the treated subjects where UC was induced. *Acinetobacter* [21], *Bifidobacterium* [22], *Clostridiaceae* [23], *Achromobacter* [24], *Burkholderia* [25], *Sutterella* [26], *Rhodococcus* [27] and *Stenotrophomonas* [28] are also observed in the microbiome of UC in humans and animal models of the disease. In our study, we confirmed the presence of microorganisms of these genera in the fecal pellets as well as cultures of DSS-induced colitis mice, with some changes in their levels associated with Bin1 mAb treatment (Fig 8). Taken together, these data specified alterations in the gut microbiome that were associated with the healthful benefits of Bin1 mAb treatment in animals subjected to DSS-induced colitis.

## Discussion

The findings of this study identify associated benefits of Bin1 mAb immunotherapy for UC treatment in preserving enteric neurons and rebalancing the gut microbiome. The identification of these effects are important, because they suggest a pleiotropic effect of Bin1 targeting in its capacity to act through multiple mechanisms to relieve UC pathophysiology.

The ENS, often referred to as a “second brain”, is large, complex and uniquely able to orchestrate gastrointestinal behavior independently of the central nervous system (CNS) [29]. The brain influences the ENS through sympathetic and parasympathetic nerves, but the bowel and its microbial content, through the ENS, also reciprocally affect the brain [7]. The movements of the small and large intestines are controlled by the ENS. Bowel movements are disrupted in UC even if small segment of the CNS is compromised. ENS abnormalities lead to irregular bowel movements and constipation [30]. Constipation is a common functional GI disorder characterized by infrequent bowel motions and/or incomplete defecation [31]. Constipation may arise due to loss of glial cells, and it points to a pathophysiological condition since the glial cells directly regulates enteric neurons and interstitial cells of Cajal through neurotrophic factors and ATP signaling [32].

We explored changes in ENS in this study because we observed that Bin1 mAb-treated animals passed more fecal pellets than control DSS-treated animals. As expected, we found that DSS treatment led to disintegrated enteric neurons; however, glial cells that protect the neurons were increased. Bin1 mAb treatment positively influenced the ENS, with neurons more similar to untreated controls than DSS-treated controls. Accordingly, an intact ENS mediated by the Bin1 mAb was associated with more normal bowel movements. Although it is known that colonic glial cells protect enteric neurons [33], as yet we have no knowledge how the glial cells cross the muscularis where the neurons are located. We demonstrated that the openings (*anigma;* Greek, opening) of the muscularis serve as a conduit for cells and molecules to transit into the muscularis. In this paper, we report changes in the ENS using microscopy techniques. We have not used any western blotting to quantitate the protein levels of the ENS.

Serotonin (5-HT) is an important neurotransmitter in the gastrointestinal tract. 95% of the body’s serotonin is produced in the intestine where it has been increasingly recognized for its hormonal, autocrine, paracrine, and endocrine actions. In the gut, a large pool of 5-HT is synthesized in the enterochromaffin (EC) cells and a smaller 5-HT pool in the ENS [34, 35]. There is functional difference between 5-HT synthesized by EC and ENS. Gastrointestinal motility depends more on neuronal than on mucosal 5-HT and that the development of dopaminergic, GABAergic, and calcitonin gene-related peptide (CGRP)-expressing enteric neurons requires neuronal 5-HT [35]. Bin1 mAb treatment altered the ENS after UC; the treatment also improved the bowel movements. We hypothesize that serotonin produced by the ENS might have influenced the bowel movements. In our further studies we will determine the neurotransmitter levels secreted by ENS upon treatment with Bin1 mAb. In addition, the food intake during DSS-treatment as well as during Bin1 mAb treatment will be monitored. The number of fecal pellets could be influenced not only by enteric neurons, but also by food intake and inflamed gut.

As yet no single microorganism has been identified as an agent responsible for UC despite the large number of gut microbiome studies and the evidence supporting the claim of microbiome involvement in the pathogenesis of the disease [36]. In our study, we observed that induction of DSS-induced colitis was associated with a change in the phenotype of the bacterial population as observed by microscopy, with rebalance of the phenotype in subjects receiving Bin1 mAb treatment. These rapid changes illustrate the plasticity of the microorganismal response to an effective therapy.

Commensal *Lactobacillus* species are common inhabitants of the natural microbiota in the human gut, and by restoring homeostasis in gastrointestinal inflammatory diseases they can exert a protective role against UC [37]. It has been previously shown that *Lactobacillus acidophilus* treatment can efficiently ameliorate DSS-induced experimental colitis in mice [38]. In our study, we observed that *Lactobacillus* was totally absent in DSS-induced colitis mice but restored to significant extent by Bin1 mAb treatment. Likewise, we observed that Bin1 mAb treatment increased gut levels of the genus *Akkermansia*, which is associated with a variety of health benefits including an alleviation of UC severity. In summary, our study demonstrated that the therapeutic benefits of Bin1 mAb treatment in DSS-induced colitis mice is associated with a preservation of enteric neurons and a rebalancing of the gut microbiome. Further studies are needed to explore the causality of these effects and whether Bin1 mAb may influence serotonin in coordinating them.

## Supporting information

S1 Data (XLSX)
